# A Principled Framework for Mendelian Randomization in Oral Health Research

**DOI:** 10.1111/jre.13411

**Published:** 2025-05-09

**Authors:** Nasir Z. Bashir, Mario Romandini, Moritz Kebschull, Konstantinos K. Tsilidis, Dominique S. Michaud, Stephen Burgess

**Affiliations:** 1 MRC Biostatistics Unit, University of Cambridge, Cambridge, UK; 2 Perio-Implant Innovation Center, Institute for Integrated Oral, Craniofacial and Sensory Research – National Clinical Research Center of Stomatology, Ninth People's Hospital, Shanghai Jiao Tong University School of Medicine, Shanghai, China; 3 Periodontal Research Group, Institute of Clinical Sciences, College of Medical & Dental Sciences, The University of Birmingham, Birmingham, UK; 4 Department of Epidemiology and Biostatistics, School of Public Health, Imperial College London, London, UK; 5 Department of Hygiene and Epidemiology, School of Medicine, University of Ioannina, Ioannina, Greece; 6 Department of Public Health & Community Medicine, Tufts University, Boston, MA, USA; 7 Cardiovascular Epidemiology Unit, Department of Public Health and Primary Care, University of Cambridge, UK

**Keywords:** Causal Inference, Epidemiology, Mendelian Randomization, Randomized Controlled Trial, Dentistry, Periodontal Diseases

## Abstract

In recent years, the popularity of Mendelian randomization (MR) as a technique to investigate causal relationships has grown exponentially. This approach leverages randomness inherent in the allocation of genetic variants to circumvent issues of unobserved confounding and reverse causation that compromise many causal claims based on observational data. In this manuscript, the key assumptions required for MR analyses to yield credible causal inferences are reviewed. It is emphasized that even technically rigorous MR analyses may yield implausible conclusions if these assumptions are violated. The goal is to provide readers from non-statistical backgrounds with a clear understanding of the foundational assumptions underpinning MR, complemented by a detailed discussion of specific considerations in oral health research, particularly when periodontitis is used as the exposure or outcome. As with any study design, MR is appropriate and can provide compelling evidence for causality in certain contexts, while its application may be unwarranted in others. This methodological article aims to equip readers with the tools to critically assess MR findings, distinguish scenarios where its use is justified, and understand the key steps for correct execution and reporting. A checklist for assessing the credibility of MR investigations is also provided.

Mendelian randomization (MR) is an epidemiological approach that uses genetic variants as instrumental variables to investigate the putative causal effect of an exposure on an outcome.^1^ Many resources are available on MR, including guidance on performing and reporting MR analyses,^2,3^ reviews tailored for the general medical audience,^4^ and discussions specific to periodontology.^5^ In this manuscript, the principles of MR are introduced, examples from periodontology are highlighted, and unique considerations when applying this method to the study of periodontitis are discussed. Datasets relevant to periodontology are also reviewed, to facilitate the application of MR in the field. The aim of this methodological article is to increase the accessibility of MR within oral health research, while equipping readers with the tools to critically evaluate the reliability of MR analyses, distinguish scenarios where its use is justified, and understand the key steps for proper execution and reporting. A checklist for assessing the credibility of MR investigations is also provided.

## What are Instrumental Variables?

**Figure 1:**
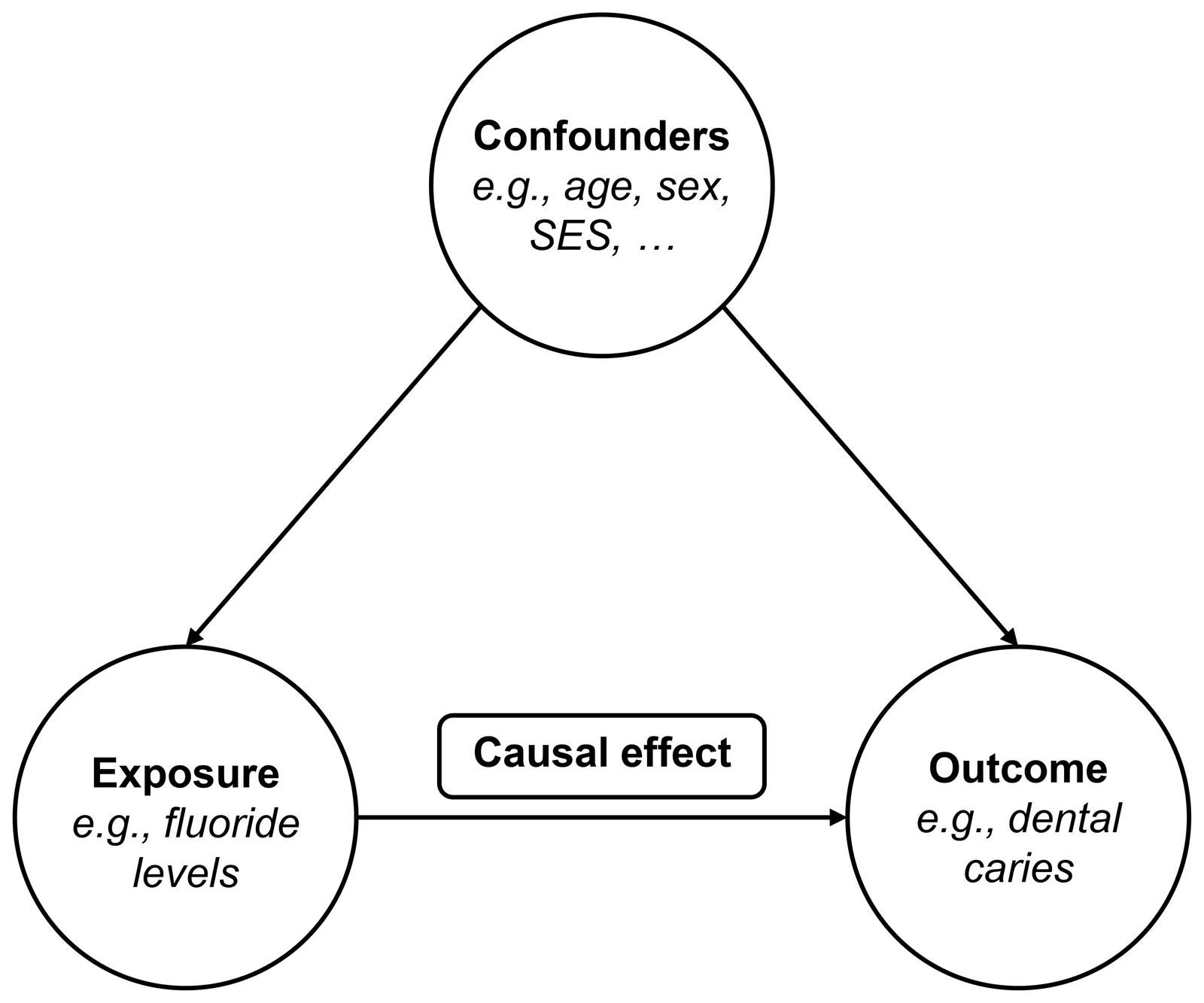
Directed acyclic graph representing the causal relationships in a hypothetical study investigating the effect of fluoride levels on dental caries. Arrows indicate that one variable causally influences another, with the direction of the arrow representing the causal relationship. The causal effect of interest is labelled in the diagram.

Suppose an investigator wants to determine whether low fluoride levels are a causal risk factor for dental caries (i.e., whether increasing fluoride levels would reduce the risk of dental caries). However, there are confounders which influence both fluoride levels and dental caries risk, such as age, sex, and socioeconomic status (Figure 1). Hence, an unadjusted analysis could show a significant association between fluoride levels and dental caries, even if no causal effect exists. Traditional analytical methods, such as multiple regression analysis, can adjust for known confounders. However, it is impossible to guarantee that all confounders are known and have been accurately measured and accounted for. In other words, even when a data analysis adjusting for known confounders is performed, observational associations are always subject to risk of residual confounding. Confounders are variables that influence both the exposure and outcome, but they are not the only source of bias. Other key sources of bias may include measurement error, selection bias, and reverse causation.

Instrumental variable analysis is a statistical technique used to uncover cause-and-effect relationships when traditional epidemiological methods might yield misleading results.^6^ An instrumental variable (or instrument) is a special type of variable that meets three assumptions:

1. It is associated with the exposure (relevance assumption),
2. It is not associated with the outcome via an uncontrolled confounding pathway (exchangeability assumption), and
3. It does not directly affect the outcome, except potentially through its effect on the exposure (exclusion restriction assumption).

The first assumption can be tested statistically by assessing whether an instrument is associated with an exposure, whilst the remaining two cannot. The second assumption implies that the genetic variant is not associated with any exposure-outcome confounder, nor are there any instrument-outcome confounders. The latter two assumptions can only be justified based on biological understanding of the selected variants. Taken together, these three assumptions are sufficient to test the null hypothesis of no causal effect of the exposure on the outcome. However, to obtain point estimates of the effect size, additional assumptions (such as monotonicity or homogeneity of the causal effect) are required.

An instrumental variable divides the population into groups with different average exposure levels while maintaining the same levels, on average, of confounding variables. Hence, any association between a valid instrumental variable and the outcome can only occur if there is a causal effect of the exposure on the outcome.^7^ A lack of association means that there is failure to find evidence of a causal effect, though a true causal effect could also go undetected due to low statistical power, which can be exacerbated by the presence of weak instruments (i.e., those which are weakly associated with the exposure). Randomized treatment allocation in a clinical trial is an example of an instrumental variable. It affects the exposure of interest, it is not associated with confounding factors, and it cannot directly influence the outcome (assuming the trial is appropriately blinded and randomization is performed correctly). However, there are other examples of plausible instrumental variables beyond investigator-instigated random allocation. In many cases, natural experiments arise, in which external circumstances divide the population into groups in a way that the division is unaffected by confounders.^8^

Returning to the previous example of an investigator assessing the causal effect of fluoride levels on the risk of dental caries. Suppose one town’s water supply naturally contains higher levels of fluoride than another town, but, in all other ways, the two towns are the same. In this case, town of residence serves as an instrumental variable, dividing the population into groups with different average fluoride levels, yet remaining similar in all other respects. This approach enables the assessment of fluoride’s causal effect on dental caries risk. Notably, this represents an example of an instrumental variable that is not a genetic variant.

## Comparison with a Randomized Trial

**Figure 2:**
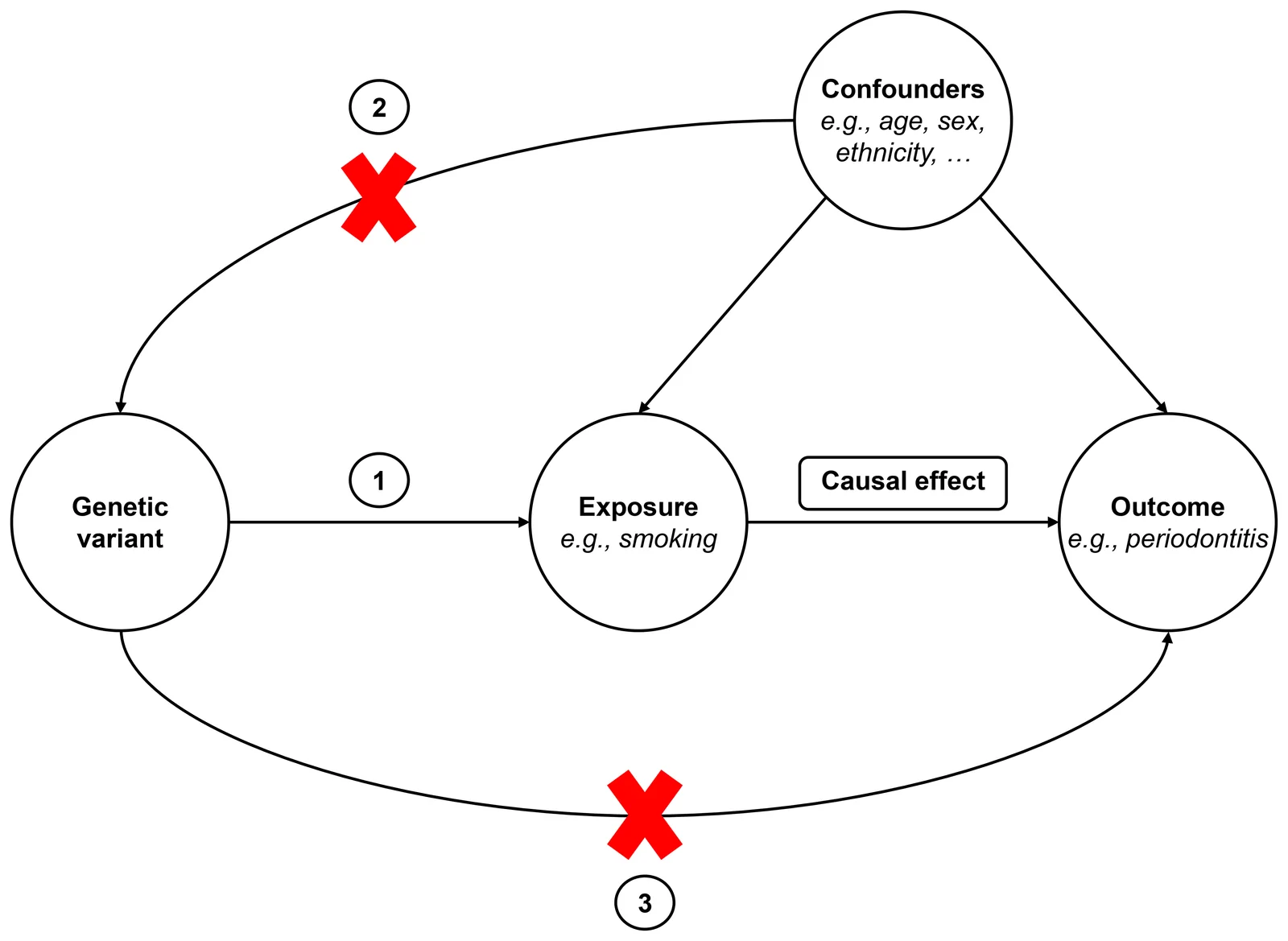
Directed acyclic graph representing the causal relationships in a hypothetical MR study investigating the effect of smoking on periodontitis. The three core assumptions required for valid MR analyses are labelled in the diagram: (1) The effect of the genetic variant on the exposure (relevance). (2) No effect of the confounders on the genetic variant (exchangeability). (3) No direct effect of the genetic variant on the outcome (exclusion restriction).

**Figure 3:**
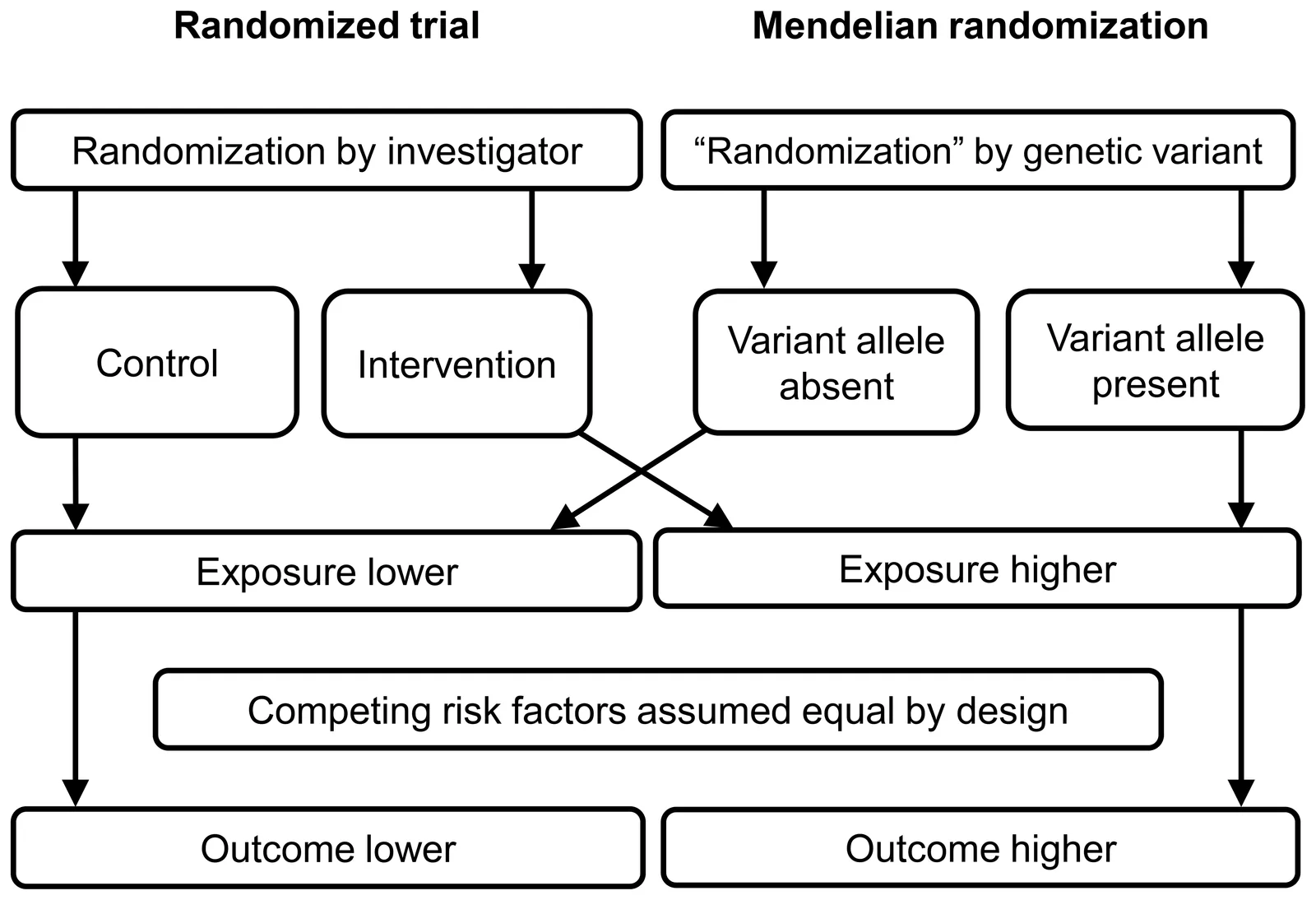
Schematic diagram showing the analogy between a simple two-armed randomized clinical trial, and an MR analysis with a dichotomous genetic variant as the instrumental variable. In both cases, under the instrumental variable assumptions, an association between the outcome and either treatment allocation (in a randomized clinical trial) or the genetic variant (in MR) is indicative of a causal effect of the exposure on the outcome.

In MR, genetic variants are used as instrumental variables and must adhere to all the three previously mentioned instrumental variable assumptions (Figure 2). Due to Mendelian inheritance, many genetic variants are distributed in the population as if they were randomly assigned; that is, they are independent of key confounding variables.^9,10^ Hence, if appropriately selected, genetic variants function analogously to randomization in a trial, dividing the population into groups with different average levels of the risk factor, but comparable levels of confounding variables (Figure 3). Furthermore, genetic variants are fixed at conception, so they cannot be influenced by confounding factors later on in life. Therefore, an association between genetically-predicted levels of the risk factor and the outcome indicates a causal effect of the risk factor on the outcome.^11^

The statistical core of an MR analysis involves assessing whether genetic predictors of a modifiable risk factor are associated with an outcome.^12^ This is analogous to a randomized clinical trial where the randomized intervention aims to modify the levels of an outcome, by raising or lowering levels of the risk factor.^13^ However, where a trial typically compares intervention versus control groups, MR compares individuals with genetically-predicted high versus low levels of the risk factor. The rationale is that, while the risk factor may be confounded in its association with the outcome, the randomized group allocation (i.e., genetic predictor) is unconfounded. However, MR should not be viewed merely as a statistical exercise. It requires a detailed understanding of biological pathways to motivate and justify the choice of genetic predictors as instrumental variables. The appropriate selection of genetic variants is critical to the reliability of an MR analysis.^14^ For findings to reflect the impact of changing a risk factor in practice, genetic variants must follow the gene-environment equivalence principle; that is, any chosen variant must influence the exposure in a way similar to a given intervention on the exposure.^15^ The key steps required for conducting an MR analysis are summarized in Box 1.

Whilst randomization and Mendelian inheritance share conceptual similarities, it would not be correct to assume that MR and randomized trials are equivalent in all aspects. First, the two approaches estimate different types of causal effects. In a randomized trial, the intervention typically has an acute effect on the exposure; that is, it induces changes for a short period of time. However, genetic variants have long-term effects; that is, they potentially affect the exposure from the time of conception. In addition, genetic variants typically have modest effects on the exposure, whilst randomized trials may administer interventions with a relatively large effect. In other words, MR is estimating the effect of a life-long, typically small, change in levels of the risk factor. On the other hand, randomized trials are estimating the effect of a short-term, potentially large, change in levels of the risk factor. Another consideration is that the MR estimate can change with time-varying exposures. For example, this means that the association between the genetic variant and the exposure might be different at age 30 than at age 60, and one would get different estimates of the causal effect depending on when the exposure was measured. Finally, despite exploiting apparent randomness in allocation of germline variants, there are situations where MR analyses can still be susceptible to bias from reverse causation.^16^

There are also ways in which MR offers advantages over randomized trials. There are some exposures for which it is unethical and/or impractical to carry out a randomized trial (e.g., cigarette smoking), but MR allows us to assess the effects of such exposures in a robust manner. Similarly, issues such as non-compliance can be circumvented since the individual has no choice in the genetic variants which they inherit. However, this is a nuanced area of discussion as genes can exert their effects in a differential manner due to factors such as gene-environment interaction.^17^

To summarize, MR uses the principles of instrumental variable analysis to allow for assessment of causal effects, but there are still differences between MR estimates and estimates obtained from a typical randomized trial.

## Common Terminology

Instrumental variable methods have a rich history which predates MR, originally being developed in the field of economics.^18^ As a result, there is a wide range of terminology which applies broadly to the field of instrumental variables, and there are some concepts which are specific to MR. Below, readers are provided with a description of some of the pertinent terminology, and a glossary summarizing the terminology is reported in Table 1.^19^

### Individual-level and summary-level analyses

Conventional observational analyses are typically performed using a single individual-level dataset. Such data contain information on all of the individual participants and there are well-established methods for carrying out instrumental variable analysis with individual-level datasets.^20^ However, genetic data is highly sensitive and strictly regulated in terms of whom it can be accessed by. Therefore, individual-level data are often unavailable, and one only has access to summary statistics, which are typically derived from a GWAS that was carried out on the individual-level data. A seminal development in MR methodology was the emergence of methods which facilitate instrumental variable analyses with summary-level GWAS data.^21^ There are further complicating factors in that some genetic datasets may measure only the exposure, and others may measure only the outcome. If the genetic variant, exposure, and outcome are all measured in a single sample, the study is said to follow a one-sample MR design. Another key advance in MR methodology was the development of the so-called two-sample design.^22^ This is the scenario where the gene-exposure association has been measured in one study, the gene-outcome association has been measured in another, and then the two are combined to obtain the MR estimate of the causal effect. Typically, it is common for two-sample designs to be used when only summary-level data are available, whilst one-sample designs are more common with individual-level data.

### Variant selection

Once the data source has been established, investigators usually consider how they are going to select genetic variants. There are two main approaches here – selection based on biological criteria and selection based on statistical criteria. In the case of biologically-driven selection, it may already be known that a genetic variant has a specific effect on the exposure of interest and would be a valid candidate to be used as an instrumental variable. Provided there is a sufficiently strong association with the exposure, one can proceed with MR analysis using this variant. Alternatively, it is possible to select variants in a manner that is based solely on statistical criteria. Most commonly, this is done by carrying out a GWAS to screen for genetic variants which are associated with the exposure. Typically, those variants which meet a genome-wide threshold of statistical significance are used as instruments.

### Biological versus statistical criteria

The question which naturally follows relates to the number of instruments one should use. If a GWAS identifies multiple instruments, then it is possible to combine them and obtain a single MR estimate, using principles from meta-analysis.^21^ It is common for studies using statistically-driven criteria to include a greater number of instruments than those which use biologically-driven selection criteria. Studies using multiple variants in this manner are referred to as polygenic analyses. On the other hand, biologically-driven selection usually results in the use of fewer instruments, often just a single instrument which has a specific effect on a target protein of interest. Such studies are referred to as *cis*-MR analyses.

**Figure 4:**
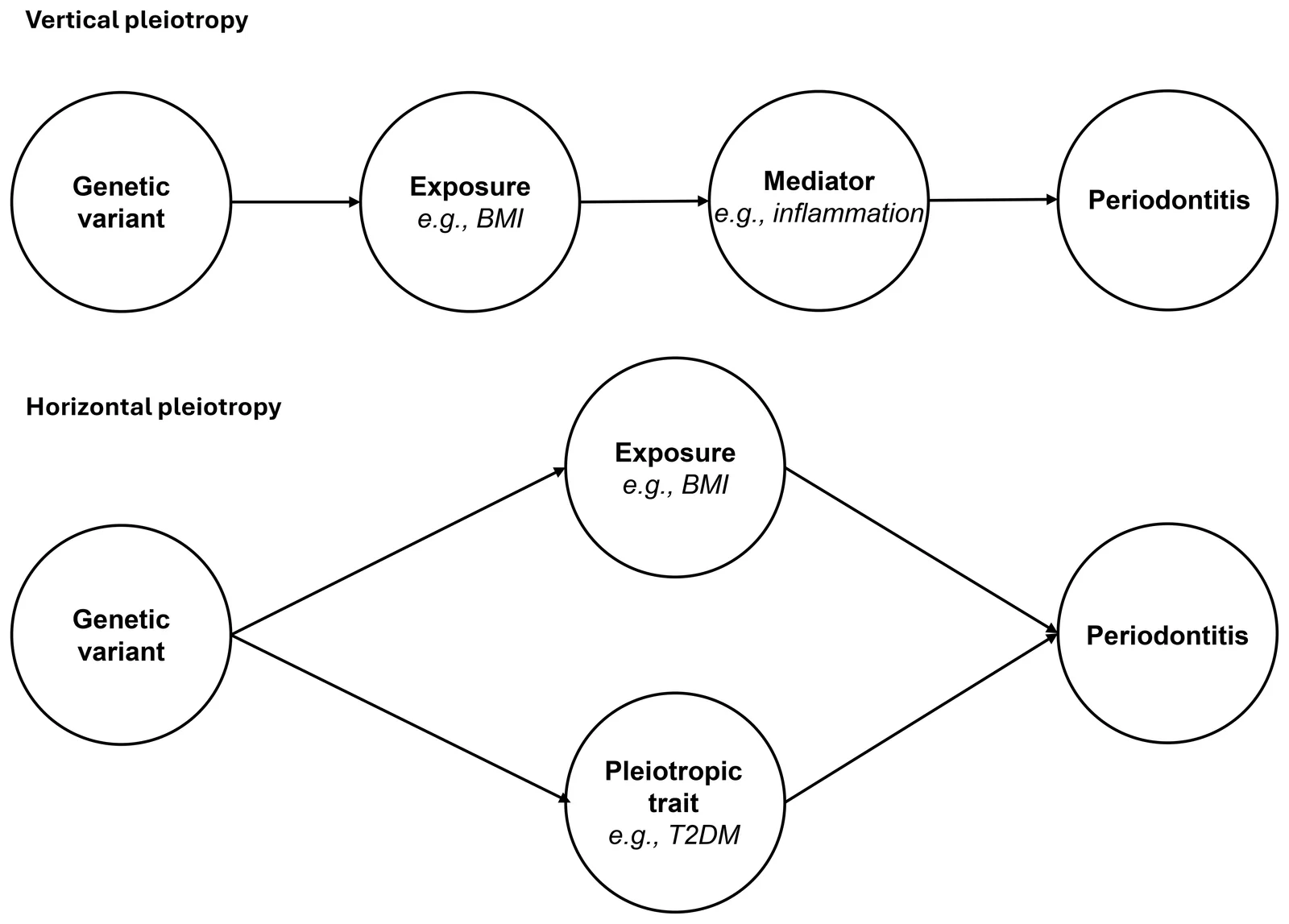
Directed acyclic graph illustrating pleiotropy in a hypothetical MR study investigating the effect of body mass index (BMI) on periodontitis. Vertical pleiotropy does not cause bias in the MR analysis as inflammation is on the causal pathway from BMI to periodontitis. Horizontal pleiotropy does cause bias as type 2 diabetes mellitus (T2DM) has effects on periodontitis via a pathway distinct from BMI, so the instrument is in violation of the exclusion restriction assumption.

The primary benefit of *cis*-MR analyses is that one can be more confident that the genetic variants are influencing the outcome solely through their effect on the exposure. When genetic variants have been identified based only on statistical criteria, it becomes more difficult to justify whether such variants are specific for the exposure, or whether they influence the outcome through other pathways. When variants influence an outcome through additional pathways, then they are in violation of the third instrumental variable assumption (exclusion restriction). Variants exerting these types of effects are said to be pleiotropic. There are two types of pleiotropy: horizontal and vertical pleiotropy. In particular, horizontal pleiotropy results in biased MR estimates (Figure 4). This should not be interpreted as suggesting that polygenic analyses are inherently flawed. Polygenic analyses come with their own benefits, such as the facilitation of sensitivity analyses, multivariable analyses, and, more generally, the ability to test hypotheses which are more complex than those which can be tested with only a single variant.^23,24^ The key point, however, is that investigators must give adequate consideration to whether the validity of their instruments is actually plausible. The reason this becomes more challenging in a polygenic MR analysis is because there are now many instruments which could potentially violate these assumptions.

### Gene-environment equivalence

As aforementioned, the three instrumental variable assumptions are sufficient to test whether an exposure has an effect on the outcome. However, this does not address clinical translation of the findings. For example, suppose investigators find that body mass index (BMI) has a causal effect on periodontitis. It remains uncertain how modifying BMI would actually affect periodontitis, because there are many different ways one can intervene on BMI (diet, exercise, medication, amongst others). In order to draw conclusions about how the causal effect would translate into clinical practice one must assume the principle of gene-environment equivalence holds true.^15^ In essence, this assumes that the effect of changing the exposure by genetic variation is equivalent to the effect of changing the exposure by environmental means. In other words, it is possible for one to identify a causal effect from an MR analysis, but there is no guarantee that the causal effect will be a clinically relevant estimate unless gene-environment equivalence holds. To emphasize why this is important – it is possible for an MR analysis to be carried out in a statistically rigorous manner with all reporting requirements met, and yet the findings of the study do not translate directly into a clinical setting as the variant does not mimic the intervention of interest.

## Non-randomness in Genetic Variation

There are several biological reasons why genetic variants may not be distributed in the population as if they are randomly assigned. If two genetic variants are located in close proximity on the genome, then they are more likely to be inherited together. In other words, it is likely that a genetic variant will be correlated with other variants which are nearby, but uncorrelated with variants which are far away. When two variants are correlated in this way, they are said to be in linkage disequilibrium (LD).^25^ The primary concern with LD is that it can lead to invalid analyses. Consider the case where there exist two genetic variants in LD; the first has an effect on the exposure and the second has an effect on a confounder. Both variants will be associated with the exposure and the confounder. Therefore, if either variant is used as an instrument, then the MR analysis is invalid due to violation of exclusion restriction.

Linkage disequilibrium is a phenomenon which occurs at the variant-level, but there are also population-level considerations which must be taken into account. Population stratification is the process by which subgroups of the population sharing a common ancestry become identifiable by their genotype.^25^ This could occur by a number of processes, such as geographic division of the population, or non-random mating.^25^ Regardless of the mechanism which leads to stratification, the end result is that the allele frequencies and genetic composition of these subgroups diverge over time. If these genetic differences correlate with both the phenotype of interest and ancestry, then this induces confounding. A classic example of this would be to imagine a behavioral scientist who tries to identify the “chopsticks gene” (i.e., a gene that predisposes individuals to eat with chopsticks); there would be an immediate issue of population stratification.^26^ They may identify variants which are significantly correlated with chopstick use and incorrectly conclude they have identified the “chopsticks gene”. The issue is that this variant likely just has different allele frequencies between European and East Asian populations where, of course, chopstick use differs due to cultural and not genetic factors.^26^ This leads to a spurious (i.e., non-causal) correlation between the genetic variant and chopstick use. One may initially dismiss such an example as ridiculous, and a mistake that no one could make. But now consider replacing “behavioral scientist” with “epidemiologist”, “chopsticks use” with “periodontitis”, and “European versus East Asian” with “European versus African American”. Now this seems like a plausible study that could produce important results. Yet, it is flawed by the exact same issue of population stratification. There are several approaches to minimize the impact of population stratification, including restricting a GWAS analysis to a clearly defined and well-mixed population, and adjustment for genomic principal components of ancestry. However, such methods are imperfect and tend to be designed for European-ancestry populations (which have distinct population groups) rather than majority world populations (which often have deeply admixed populations). Additionally, such approaches prioritize analyses in European-ancestry populations, as these have the largest available sample sizes. Accounting for population structure in a way that maintains validity but allows analyses in underrepresented groups is a topic of current research.^27–29^

## The Key Issue: Plausibility of the Causal Claims

**Figure 5:**
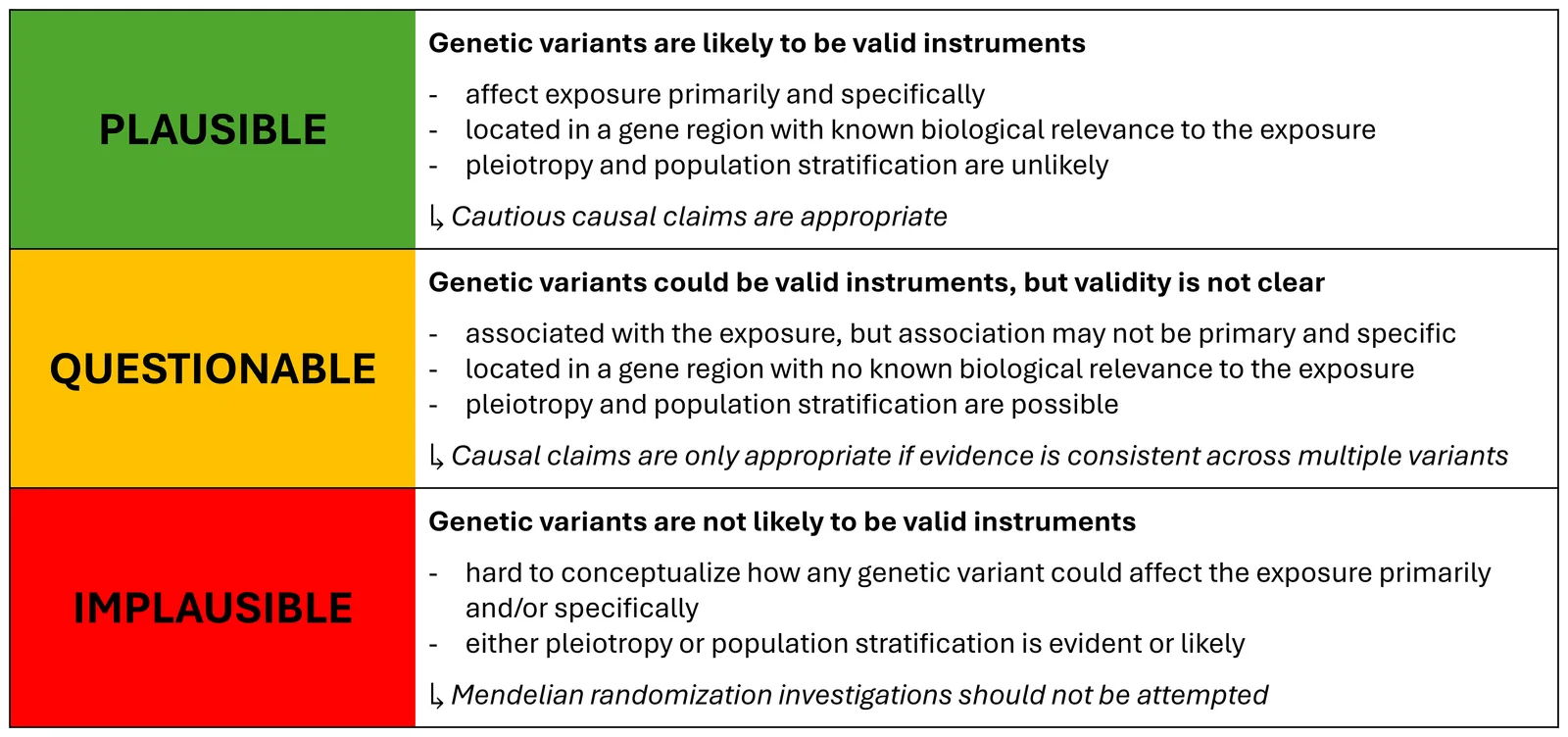
Three categories of MR investigations: plausible, questionable, and implausible.

MR studies can be broadly categorized into three groups: plausible, questionable, and implausible. The category which an investigation falls into depends on the plausibility of the instrumental variable assumptions being met by the selected genetic variants (Figure 5).

The most plausible MR studies are when the exposure is the concentration of a protein, as one can be more confident that the analysis is valid.^30^ In this case, genetic variants within the protein-coding gene region are plausible instrumental variables: they behave like a randomized intervention on the exposure. For example, genetic variants in the *CRP* gene region are specifically associated with C-reactive protein levels, affecting C-reactive protein levels directly (rather than indirectly through a long causal pathway)^31^. For a variant in the *CRP* gene region that influences C-reactive protein levels, it is plausible that any genetic association with the outcome is due to a causal effect of C-reactive protein. In other instances, the exposure may not be the level of a single protein, but there is a clear biological relevance for the variants to the exposure, making it plausible that the genetic variants influence the exposure primarily and specifically.

Some genetic variants are pleiotropic, meaning they affect multiple distinct causal pathways. Alternatively, genetic associations may arise due to population stratification.^32^ In either case, the instrumental variable assumptions are violated, and a genetic association with the outcome could have an alternative explanation, other than being an effect of the exposure. For example, a GWAS has identified associations between variants in the *ABO* gene region and various gut microbiota.^33^ However, the *ABO* gene is involved in regulating multiple biological processes, so these genetic associations are not specific to the gut microbiome. Therefore, it would be unreasonable to perform an MR analysis to assess the effect of the gut microbiome using variants in the *ABO* gene region, as such variants are likely to be pleiotropic.

In some cases, it is plausible that genetic variants primarily and specifically influence the exposure, but the biological pathways by which particular genetic predictors act remains unknown. This uncertainty undermines the reliability of an MR analysis using these variants. However, if several genetic variants from different gene regions all associate with the exposure, then consistent associations with the outcome would represent evidence for a causal effect of the exposure, even if the MR assumptions are questionable.^34^ For example, biological mechanisms affecting sleep duration are unclear, but several genetic predictors of sleep duration have been discovered.^35^ If multiple genetic predictors of sleep duration associate with a disease outcome, while it is possible that all the associations arise from pleiotropy or population stratification, a causal effect of the risk factor is a more parsimonious explanation. Such analyses are often worth performing, as they can provide valuable supporting evidence for the causal nature of the exposure. However, investigators are encouraged to prioritize MR studies using biologically relevant genetic variants, and to consider biologically agnostic analyses either as supplementary analyses, or if biologically informed analyses cannot be attempted.

In an MR study of coffee consumption and periodontitis, the authors concluded that there was evidence supporting a harmful causal effect of coffee consumption on risk of periodontitis.^36^ However, the biological evidence for the relevance of the genetic variants used as instruments for coffee consumption is limited, and a systematic review of observational studies showed no evidence of an association between coffee consumption and periodontitis.^37^ Additionally, robust MR sensitivity analyses employed by the authors did not provide evidence supporting a causal effect. Indeed, further examination of the genetic variants used as instruments highlights that they may have effects unrelated to coffee consumption (rs1057868: complete blood count measures; rs2472297: body mass index; rs66723169: leukocyte count). In such cases, while the analysis may be worth performing, caution is warranted when interpreting the findings as providing substantial evidence for a causal effect.

Finally, there are exposures for which it is implausible that genetic variants could act as valid instrumental variables. An example is exposure to air pollution, which is determined by an individual’s environment and not their genetic make-up.^38^ Conceptually, it seems unlikely that there are genetic variants that could satisfy the gene-environment equivalence principle. That is, a genetic variant that raises circulating levels of pollutants is likely to have pleiotropic effects on the outcome. Therefore, MR analyses for such exposures are typically unreliable and should generally be avoided.

This illustrates the difficulty in performing and interpreting published MR analyses. Depending on the choice of variants, an analysis may provide reasonable evidence for a causal effect, or it may not offer any reliable evidence at all. Alternatively, it may be that analysts have conducted the analysis to the best of their ability, but it only provides supportive evidence for a causal effect. While the choice of variants is not the only consideration in judging the reliability of an MR study, it is the most fundamental. An analysis can follow all available guidelines perfectly, but, if the genetic variants are not plausible instruments, then the analysis has little value. Investigators conducting MR studies in the field of periodontology will typically either treat periodontitis as the exposure or the outcome in the analysis. Both scenarios present unique challenges.

## Periodontitis as the Exposure

Periodontitis is an inflammatory disease that, like other chronic diseases, arises from a combination of biological, socio-economic, and lifestyle factors.^39–42^ Most genes exert their effects by modulating protein expression; therefore, many of the genetic predictors of periodontitis likely affect proteins that are pertinent to periodontal health, such as those that regulate the inflammatory cascade.^43,44^ For these variants, investigators need to assume that changes in protein levels and changes in periodontitis risk have equivalent downstream effects. For instance, to use a genetic variant in the *TNF* gene region that encodes tumor necrosis factor-alpha (TNF-α), it would be necessary to assume that perturbation of circulating TNF-α can only affect the outcome via periodontitis. However, TNF-α does not only affect periodontal health, but also plays a role in insulin resistance, rheumatic diseases, and inflammatory bowel disease.^45,46^ Therefore, it would not be possible to differentiate the effect of periodontitis on a given outcome from the effect of all the other diseases which are also downstream of TNF-α. In other words, the causal pathway from the genetic variant to the outcome is not specific, in that it passes through multiple diseases, i.e., the variant is pleiotropic.

This highlights a fundamental problem in using periodontitis as an exposure. The variants which are candidate instrumental variables primarily affect protein expression, and periodontitis—to the best of current established knowledge—is far downstream from protein expression. Therefore, if a variant is used to instrument periodontitis directly, it is unlikely that exclusion restriction (or gene-environment equivalence) is met, as there are many different pathways through which that variant may act. Instead, researchers may be tempted to identify genetic variants that influence some of the socio-economic or lifestyle factors that affect periodontal health, such as smoking or oral hygiene habits. However, additional issues are encountered in this case. It is indeed implausible to find genetic variants that affect lifestyle factors in a way that is specific to periodontitis. For example, if a variant is identified that is strongly associated with poor oral hygiene habits, how can one be certain that this variant is not more broadly associated with unhealthy lifestyle choices, rather than specifically oral hygiene habits? And if a gene predisposes individuals to generally make unhealthy lifestyle choices, then this would open many pleiotropic pathways. Once again, this leaves a scenario where elucidating the causal effect of periodontitis through MR is challenging.

It is conventional for genetic variants used in MR to be associated with the exposure at the genome-wide level of significance, although this requirement may be relaxed if there is strong biological rationale for considering variants in a particular gene region. While the heritability of periodontitis is relatively high,^47^ very few variants have been independently associated with periodontitis at the genome-wide level of significance,^48^ and replication studies have struggled to reproduce these genetic signals^5^. While this may result from the use of heterogenous and/or inaccurate case definitions, MR studies using periodontitis as the exposure may have insufficient power to detect a causal effect. Combined with the fact that the gene-environment equivalence principle is required to infer the effect of periodontitis on a given outcome (as opposed to an effect of liability to periodontitis^49^), it becomes very difficult to conceive how periodontitis can reliably be used as the exposure in an MR analysis. This is not to say it is impossible; on numerous occasions, instrumental variables have been used in creative ways to estimate causal effects.^50,51^ The problem being outlined, however, is that it is not clear how this can be done in a reliable manner using only genetic instruments for periodontitis. Indeed, MR analyses using periodontitis as the exposure may still be of interest when it is the only study design available, but investigators should use appropriate language when stating the strength of their causal claims.

## Pleiotropy-robust Methods: A Possible Solution?

The immediate answer to the concern raised above is to deploy one of the many pleiotropy-robust methods which have been developed. The use of such methods is important in polygenic MR analyses.^52^ However, pleiotropy-robust methods themselves make assumptions which also seem unlikely to hold true in the case of periodontitis as an exposure. Full treatment of pleiotropy-robust methods for MR is given elsewhere, but here the four most widely-used methods are summarized and it is explained why they do not completely address the problems brought to light earlier on.

The weighted median method is a simple but versatile estimator for MR analyses, in that it allows up to half of the selected variants to be potentially invalid instruments (known as the majority valid assumption).^53^ Herein lies the issue; it is difficult to identify even a single valid instrument for periodontitis, so it seems very difficult to justify that at least half of the instruments in a given analysis are truly valid. When this assumption is violated, then the method will still yield biased estimates of the causal effect.

The mode-based estimator shares a similar problem. It assumes that, if there are groups of genetic variants with different estimates of the causal effect (i.e., some pleotropic and some valid), then it is the group which contains the largest number of variants that is estimating the true effect (known as the plurality valid assumption).^54^ Again, it is hard to identify even a single valid instrument for periodontitis, so it seems very difficult to justify the plurality valid assumption.

The MR-PRESSO method removes genetic variants which give outlier effects and then assesses how the removal of these outliers changes the causal estimate.^55^ The same issue arises again, if the majority of selected variants are invalid then it could easily be the case that the valid instrument(s) are identified as outliers and removed. Furthermore, MR-PRESSO is known to be prone to greatly inflated type I error rates when several invalid instruments are present, which seems to be the most likely scenario when attempting to identify variants for periodontitis.^52^

The MR-Egger method treats the effect estimate from each genetic variant as its own study and then uses Egger regression, a method from meta-analysis, to detect bias from pleiotropy.^53^ The MR-Egger method hinges on the so-called InSIDE assumption which essentially states that the size of the pleiotropic effect for a genetic variant is uncorrelated with the size of its association with the exposure. The InSIDE assumption is known to be often unrealistic, which results in increased error rates when it is violated.^24^ Having said that, specifically for periodontitis, there is no empirical evidence to strongly support or refute the InSIDE assumption.

As highlighted above, there are many pleiotropy-robust methods available to investigators, but it is not true that they perform equally well for all choices of exposure. To overcome the issues pertaining to the use of periodontitis as an exposure, careful thought is required regarding the choice of instruments, and justification that such a variant can truly meet the assumptions required for a valid MR analysis. It is emphasized that the use of pleiotropy-robust methods is *not* being discouraged. They should be routinely used, but, investigators should be aware that they are not an infallible solution to the problem of pleiotropy.

## Periodontitis as the Outcome

Assessing periodontitis as the outcome is generally more amenable to MR analysis, but researchers must take into account several considerations. Periodontitis is clinically defined by the presence of clinical attachment loss (CAL), which is often complemented by additional parameters to differentiate stage, grade, and extent of the disease.^56^ These parameters may include probing pocket depth (PPD), mobility, periodontitis-related tooth loss, and masticatory dysfunction. CAL should ideally be assessed through a full-mouth examination protocol (i.e., six sites per tooth, for all 28 teeth, excluding third molars). Partial-mouth examination protocols, while more convenient, have limited diagnostic accuracy and may result in information bias.^57^ If the “true” periodontal diagnosis is ascertained by measuring full-mouth CAL and PPD, then using a partial-mouth protocol to classify individuals will weaken the association between the genetic variant and the “true” diagnosis. In effect, this is a form of measurement error. Similarly, datasets relying solely on PPD to assess periodontitis may also introduce bias. Although PPD is easier to measure, it can change following periodontal therapy (as can CAL, albeit less drastically than PPD).^58^ Radiographic bone loss, however, may serve as a reasonable surrogate for periodontitis in the absence of full-mouth CAL assessments.^59^

Another important consideration arises when a dataset relies not on clinical or radiographic measures, but on self-reported assessments, for instance derived from common questionnaire items such as, “Have you ever been told you have bone loss around your teeth?” or “Do you have any loose teeth?”. The sensitivity and specificity of these questions must be considered, as they are more likely to identify advanced cases (i.e., stage IV periodontitis). Investigators using consortium datasets that combine patients diagnosed with clinical measures and others with self-reported measures should carefully make an *a priori* decision about their exposure definition. There is a tradeoff between the increased statistical power from including all individuals in a consortium dataset versus the potential reduction in clinical relevance induced by including data from those diagnosed by self-reported measures. Additionally, this choice may potentially also affect the demographic composition of cases, as has been observed in studies of other diseases.^60^

Finally, researchers may be tempted to combine distinct oral diseases, such as periodontitis and caries, into a single “oral health” group. While these conditions share some common risk factors, they are biologically distinct diseases with separate causal pathways.^61,62^ Combining them into a heterogenous grouping diminishes the clinical interpretability of causal effect estimates. Although grouping outcomes may increase statistical power, this approach sacrifices plausibility and interpretative value, rendering the investigation less meaningful. For these reasons, combining disparate oral health outcomes is strongly discouraged.

## Datasets for Periodontitis

A wide range of datasets has been used to investigate the genetic basis of periodontitis; however, the disease definition varies between studies, and most provide only summary-level data publicly.^48^ The Gene-Lifestyle Interactions in Dental Endpoints (GLIDE) consortium is one of the most commonly utilized sources of data.^63^ On October 28 2024, MEDLINE via PubMed was searched using the search terms “mendelian randomization” AND “periodont*” and retrieved 167 results. Of these, 85 articles cited the GLIDE consortium as one of their primary sources of data. In other words, over half of the MR studies in periodontology are based on a single dataset. In addition, when considering the articles that cite any of the individual studies included in the GLIDE meta-analysis (e.g., UK Biobank or FinnGen), then the overwhelming majority of publications are derived on either GLIDE or its constituent studies.

This poses several challenges. First, the GLIDE consortium combines data from self-reported and clinical measures of periodontitis, as well as hospital registries. Several studies based on clinical measures employed partial-mouth recording protocols (e.g., SHIP) and/or PPD assessments (e.g., TwinGene). The self-reported measures are also heterogenous. These limitations are acknowledged by the consortium members^63^, but are not necessarily recognized by all authors who use the GLIDE data to carry out MR studies. Investigators do sometimes choose to restrict their analyses to the GLIDE subset of approximately 45,000 clinically defined cases and controls. This is a useful approach in that it ensures the case definition is based on clinical criteria but does come at the cost of a reduced sample size. Another factor to consider is that the available GWAS in the GLIDE consortium are limited to European populations, so it is not clear how the results based on these data will generalize to other populations.

Investigators may turn to datasets other than GLIDE, but there are substantial limitations regarding the quality of genetic data on periodontitis. A recent systematic review identified 15 GWAS on periodontitis published between 2010 and 2023.^48^ Of these, 10 studies had a sample size of fewer than 10,000 participants. Across the studies, a total of 11 variants were significant at the genome-wide level, but none of these were consistently identified across studies (although some gene regions were reported in multiple studies). This inconsistency may stem from the extensive heterogeneity between studies, especially in defining periodontitis. Even among studies using clinical measures, different examination protocols and disease definitions were employed. This highlights the urgent need for more robust GWAS, thereby enabling more reliable inferences from genetic analyses.The Million Veteran Program is an example of a large-scale study, with 2,068 traits measured in over 600,000 participants.^64^ Amongst these traits, periodontitis is included, defined according to Phecodes. Phecodes are generated by mapping a phenotype to its ICD-9 or -10 codes in the electronic health records for each patient and identifying at least two instances of such codes. Depending on whether acute or chronic periodontitis (or both) are used, up to 60,000 cases have been identified. It is currently not understood how accurately these Phecode-based diagnoses classify periodontal disease according to modern case definitions. Table 2 summarizes the current major GWAS datasets which are relevant to the study of periodontitis, based on a recent systematic review updated here with a search to account for studies published from 2023 onwards.^48,63–81^

## Examples of Good Practice

By carefully addressing its inherent strengths and limitations, MR offers a powerful framework for investigating putative causal relationships in a meaningful and robust manner. As aforementioned, the principle of gene-environment equivalence is central to the MR paradigm. A recent study by Liu et al. exemplifies this principle through their investigation of genetically proxied drug targets and their association with oral health.^86^ In their analysis, the authors selected instrumental variables supported by blood cis-expression quantitative trait loci (*cis-eQTLs*), focusing on variants within the gene regions encoding the protein drug targets. By doing so, the study ensured that genetic perturbations of the protein targets were more likely to produce downstream effects analogous to environmental perturbations. However, the authors prudently acknowledged the limitations of their findings, citing the paucity of robust data for thoroughly examining drug targets in periodontitis. Additionally, the estimated effect sizes for non-periodontal oral health outcomes were small, raising questions about their clinical relevance.^86^

The credibility of MR findings is strengthened when analyses are triangulated with complementary sources of evidence to address a given research question.^87^ For instance, there is substantial mechanistic and epidemiological evidence implicating increased blood glucose and glycated hemoglobin levels in the pathogenesis of periodontitis.^88^ An MR study corroborated such well-established findings, providing an added layer of validation.^89^

MR is particularly important in assessing exposures for which randomized clinical trials are not feasible. For example, tobacco smoking and alcohol consumption are widely recognized as risk factors for periodontitis, supported by mechanistic studies and observational research.^90,91^ However, the potential for residual confounding in these observational studies necessitates caution. Performing an RCT where individuals are assigned to smoke cigarettes or increase their alcohol consumption is both unethical and impractical. In this context, MR is particularly useful. Interestingly, non-genetic instrumental variable studies have also identified a causal effect of smoking.^92,93^ Similarly, socioeconomic status is another factor consistently implicated in the pathogenesis of periodontitis through observational studies.^94^ While RCTs to directly test this hypothesis are infeasible, MR can provide valuable evidence supporting the causal role of socioeconomic determinants, such as educational attainment, in periodontitis risk.^95^ This is feasible because genetic variants associated with biologically relevant traits for this exposure are available. While this represents a “questionable” investigation—since these variants are unlikely to influence educational attainment through a singular, well-defined mechanism—the convergence of consistent evidence across multiple variants strengthens the plausibility of a causal claim.

In summary, it is possible to conduct an MR analysis that is statistically robust, well-documented, and reported in sufficient detail to allow reproducibility, yet still lacking credibility if biological plausibility is weak. Table 3 presents a concise summary of the essential criteria to assess the credibility of an MR study.^96^ This should be combined with reporting criteria, such as the STROBE-MR guidelines, to aid investigators in carrying out analyses which are both biologically plausible and statistically sound.^3^

## Conclusions

The use of MR to investigate causal hypotheses is gaining traction in oral health research, especially in periodontology. The paradigm offers a powerful approach to circumvent the challenges of unobserved confounding that often undermine conventional observational studies. However, the validity of MR findings hinges on the fulfillment of several key assumptions. In particular, the lack of instrument validity (and, hence, gene-environment equivalence) poses significant challenges when assessing periodontitis as an exposure, rendering such analyses methodologically problematic. Within the limitations of the currently available data, evaluating periodontitis as an outcome appears to be more amenable to MR analyses, provided that valid instruments exist for the exposure of interest. Importantly, MR results are most valuable when interpreted in the context of a broader body of evidence. Triangulating findings from MR studies with results from other methodological approaches enhances the reliability of conclusions, providing a more comprehensive understanding of causal relationships.

## Supplementary Material

Box 1

## Figures and Tables

**Table 1:** Glossary of key terms.

MR Assumptions	
Relevance assumption	The chosen genetic variant(s) must be robustly associated with the exposure of interest, typically at a genome-wide significance level (p < 5 × 10^−8^).
Exchangeability assumption	The genetic variant(s) must be independent of factors that affect the outcome on alternative pathways. More precisely, there must be no confounding pathways between the variant(s) and the outcome.
Exclusion restriction assumption	The genetic variant(s) may only affect the outcome through the exposure under investigation.
**Key Concepts**	
Horizontal pleiotropy	When a genetic variant influences the outcome independently of the exposure under investigation, that is, violation of the exclusion restriction assumption. This assumption is the focus of most sensitivity analysis methods (e.g., MR-Egger, weighted median/mode). However, these methods should not be used as a replacement for careful selection of variants (or otherwise application of critical reasoning).
Vertical pleiotropy	When a genetic variant is associated with multiple traits on the same causal pathway to the outcome. This chain of traits to the outcome is what MR sets out to estimate and is not a source of bias.
Population stratification	When there are subgroups in the population that have both different phenotypic distributions and different allele frequencies for genetic variants that might be used in MR. This can result in spurious associations between genotype and phenotype.
Assortative mating	When individuals choose their partners non-randomly, for example, taller women tending to partner with taller men. Again, this can result in spurious associations between genotype and phenotype.
Dynastic effects	Dynastic or intergenerational effects occur when parental genotype affects offspring outcomes causal pathways independent of the offspring phenotype. For example, when more educated parents support their children's education. This can result in associations between genotype and phenotype that are not fully spurious, but do not occur due to the phenotype in the individuals in the sample population.
Linkage disequilibrium	Correlation (non-random association) between genetic variants due to their physical proximity on a chromosome. This can result in spurious associations between genotype and phenotype. Additionally, MR estimates from each genetic variant are pooled analogously to study estimates in a meta-analysis; therefore, naïvely combining estimates from variants that are not independent (in linkage disequilibrium) is analogous to including a trial in the meta-analysis more than once. Independent genetic variants are often selected by ‘clumping’, whereby the most significant variant is selected to represent the locus.
**Statistical Methods**	
Summary data MR	MR analysis which is carried out with aggregate statistics from GWAS data (i.e., pre-computed estimates of the gene-exposure and gene-outcome regression associations).
Individual-level data MR	MR analysis which is carried out with the raw genetic and phenotypic (i.e., exposure and outcome) data for every individual in the sample.
F statistic	The strength of association between the genetic variant and exposure, and an indicator of the (weak instrument) bias that is likely to occur in estimating the exposure-outcome association.
Two-sample MR	MR analysis where gene-exposure and gene-outcome associations are derived from different non-overlapping samples from the same underlying population. Two-sample MR analyses are typically performed using summary level data (e.g., beta-coefficients and standard errors for each genetic variant). The ability to mix and match exposure and outcome genetic association data has made two-sample MR by far the most common approach used in MR studies.
One-sample MR	MR analysis using data on the genetic variants, exposure, and outcome from the same sample. One-sample MR analyses are typically performed using individual-level data.

This table is adapted from Zhao, Burgess.^19^

**Table 2:** Summary of major GWAS datasets in periodontology.

Reference	Location	Sample size		Periodontitis type	Periodontitis definition
		Cases	Controls		
de Almeida (2024)	Germany, Netherlands, Spain	1306	7817	Aggressive	EFP-AAP^82^
Bevilacqua (2018)	Italy	442	160	Chronic	AAP^83^
de Coo (2021)	Spain	441	1141	Aggressive	EFP-AAP^82^
Divaris (2013)	Europe, US	3251	1909	Chronic	CDC-AAP^84^
Feng (2014)	Brazil, US	712	1973	Chronic	≥ 30% teeth with ≥ 5mm CAL
Hong (2015)	South Korea	414	263	Periodontitis	CDC-AAP^85^
Kurki (2023)	Finland	50000^a^	280000^a^	Chronic & acute	ICD codes
Munz (2017)	Germany, Netherlands, Turkey	2067	8819	Chronic & aggressive	Chronic: According to number of proximal sites with ≥ 4mm CAL Aggressive: ≥ 2 teeth with at least 30% alveolar bone loss, aged below 35 years, no diabetes
Munz (2019)	Europe, Germany, Netherlands, US	5095	9908	Chronic & aggressive	Chronic (European & American): CDC-AAP^82^ Chronic (German): Subjects within first and third tertile of proportion of proximal sites ≥ 4mm CAL contrasted post-stratification Aggressive (Dutch): ≥ 2 teeth with ≥ 30% alveolar bone loss, aged below 36 years
Nogawa (2024)	Japan	7059	38466	Periodontitis	Self-reported
Sanders (2017)	US	15337^b^		Chronic	CDC-AAP^85^
Schaefer (2010)	Germany, Netherlands	438	1320	Aggressive	Localized: ≥ 50% bone loss at 2 to 6 teeth Aggressive: ≥ 50% bone loss at ≥ 7 teeth
Shaffer (2014)	US	176^c^	497^c^	Chronic	Two or more sextants with probing depths of ≥ 5.5mm, then further subdivided
Shimizu (2015)	Japan	2760	15158	Periodontitis	Various AAP^83,84^
Shungin (2019)	Many	36332	470262	Periodontitis	Biobank: Self-reported GLIDE: - ARIC, SHIP, SHIP-TREND, HCHS/SOL, TwinGene, MDC, BBJ, TMDUAGP: Varying criteria, all clinical diagnoses - >WGHS: Self-reported
Tegelberg (2021)	Finland	3245^d^		Periodontitis	According to number of teeth with ≥ 4mm pockets
Teumer (2013)	Germany	4032^e^		Chronic	Four different case definitions, all clinical diagnoses
Verma (2024)	US	59952^f^	545372^f^	Chronic & acute	PhenoCodes (ICD-based)

a Estimates of up to how many cases can be included, based on FinnGen registry data. However, applied studies typically use up to 30000 cases due to specific ICD codes and QC criteria. Similarly, applied studies typically use up to 280000 controls.

b Cases and controls combined, across discovery and replication datasets.

c Upto this number, depending on definition.

d Cases and controls combined.

e SHIP and SHIP-TREND combined (differing prevalence of controls).

f Upto this number, based on maximum number of cases and controls in dbGap data file.

AAP: American Academy of Periodontology. CAL: Clinical attachment loss. CDC: Centers for Disease Control and Prevention.

**Table 3:** Checklist for assessing the credibility of a MR study.

**1.**	**Is the research question addressable using Mendelian randomization?** Is it plausible we can find genetic variants that satisfy the gene-environment equivalence assumption?
**2.**	**Is the choice of genetic variants appropriate?** Is the biological relevance of the variants to the exposure known? If chosen based on GWAS of an external dataset, how was that GWAS conducted? How was the exposure defined and measured?
**3.**	**Have the researchers sufficiently interrogated the findings?** Have approaches that assess the robustness of findings been performed? Have results been reported clearly, accounting for multiple testing if appropriate?
**4.**	**Have the findings been appropriately interpreted?** Are conclusions proportionate to our confidence in the validity of the instruments? Have alternative causal pathways from the variants to the outcome been considered?
**5.**	**Are results adequately compared to literature?** Is there sufficient discussion about coherence of findings from different approaches? Is the overall conclusion presented in a triangulation framework?

This table is reproduced from Burgess, Woolf, Mason, Ala-Korpela, Gill.^96^

## Data Availability

Data sharing is not applicable to this article as no datasets were generated or analyzed during the current study.

## Box 1 A summary of the steps involved in a MR study (adapted from Burgess et al.).^2^

**1. Define the motivation and scope**

     -     State clearly the exposure(s) and outcome(s), and the causal hypotheses to be tested

**2. Choose data sources**

     -     Identify appropriate datasets for selecting genetic variants, and estimating associations with the exposure(s) and outcome(s)

**3. Select genetic variants**

     -     Identify genetic variants associated with the exposure that plausibly satisfy the core instrumental variable assumptions (relevance, exchangeability, and exclusion restriction)

**4. Harmonize genetic data (for two-sample investigations)**

     -     Ensure genetic variants are consistently aligned across datasets for exposure and outcome, accounting for allele frequency and strand orientation

**5. Perform primary analysis**

     -     The primary analysis represents the initial assessment of evidence for a causal effect of the exposure on the outcome

**6. Conduct sensitivity analyses**

     -     Sensitivity analyses are performed to “stress test” the primary analysis result: to see how robust it is to various conditions, including violations of the instrumental variable assumptions (for example, alternative statistical methods, analyses excluding certain genetic variants, colocalization, assessment of positive/negative controls)

**7. Explore supplementary analyses**

     -     Supplementary analyses are performed to better understand the nature of the causal relationship (for example, is it similar in different populations or population subgroups?)

**8. Present findings transparently**

     -     Data and results should be presented with enough information to allow readers to assess the plausibility of the analysis and its assumptions

**9. Interpret results with appropriate caution**

     -     Results should be presented and interpreted with appropriate caution, and compared with evidence from the wider literature in a triangulation framework

## References

[1] Emdin CA, Khera AV, Kathiresan S (2017). Mendelian Randomization. JAMA.

[2] Burgess S, Davey Smith G, Davies NM (2020). Guidelines for performing Mendelian randomization investigations. Wellcome Open Research.

[3] Skrivankova VW, Richmond RC, Woolf BAR (2021). Strengthening the reporting of observational studies in epidemiology using mendelian randomisation (STROBE-MR): explanation and elaboration. BMJ.

[4] Davies NM, Holmes MV, Davey Smith G (2018). Reading Mendelian randomisation studies: a guide, glossary, and checklist for clinicians. BMJ.

[5] Haworth S, Timpson NJ, Divaris K (2024). Mendelian randomization studies of periodontitis: Understanding benefits and natural limitations in an applied context. Journal of Clinical Periodontology.

[6] Martens EP, Pestman WR, de Boer A, Belitser SV, Klungel OH (2006). Instrumental variables: application and limitations. Epidemiology.

[7] Greenland S (2000). An introduction to instrumental variables for epidemiologists. International Journal of Epidemiology.

[8] Craig P, Katikireddi SV, Leyland A, Popham F (2017). Natural Experiments: An Overview of Methods, Approaches, and Contributions to Public Health Intervention Research. Annual Review of Public Health.

[9] Davey Smith G, Lawlor D, Harbord RM, Timpson N, Day I, Ebrahim S (2007). Clustered environments and randomized genes: a fundamental distinction between conventional and genetic epidemiology. PLoS Medicine.

[10] Taylor M, Tansey KE, Lawlor DA (2017). Testing the principles of Mendelian randomization: Opportunities and complications on a genomewide scale. bioRxiv.

[11] Didelez V, Sheehan N (2007). Mendelian randomization as an instrumental variable approach to causal inference. Statistical Methods in Medical Research.

[12] Lawlor DA, Harbord RM, Sterne JA, Timpson N, Davey Smith G (2008). Mendelian randomization: using genes as instruments for making causal inferences in epidemiology. Statistics in Medicine.

[13] Thanassoulis G, O'Donnell CJ (2009). Mendelian randomization: nature's randomized trial in the post-genome era. JAMA.

[14] Stephen B, Héléne Toinét C (2024). Incorporating biological and clinical insights into variant choice for Mendelian randomisation: examples and principles. eGastroenterology.

[15] Sanderson E, Glymour MM, Holmes MV (2022). Mendelian randomization. Nature Reviews Methods Primers.

[16] Burgess S, Swanson SA, Labrecque JA (2021). Are Mendelian randomization investigations immune from bias due to reverse causation?. Eur J Epidemiol.

[17] Keller MC (2014). Gene × environment interaction studies have not properly controlled for potential confounders: the problem and the (simple) solution. Biol Psychiatry.

[18] Angrist JD, Pischke J-S (2009). Mostly Harmless Econometrics: An Empiricist’s Companion.

[19] Zhao SS, Burgess S (2024). Use of Mendelian randomization to assess the causal status of modifiable exposures for rheumatic diseases. Best Practice & Research Clinical Rheumatology.

[20] Angrist J, Imbens G (1995). 2-stage least-squares estimation of average causal effects in models with variable treatment intensity. Journal of the American Statistical Association.

[21] Burgess S, Butterworth A, Thompson SG (2013). Mendelian Randomization Analysis With Multiple Genetic Variants Using Summarized Data. Genetic Epidemiology.

[22] Pierce BL, Burgess S (2013). Efficient Design for Mendelian Randomization Studies: Subsample and 2-Sample Instrumental Variable Estimators. American Journal of Epidemiology.

[23] Burgess S, Thompson SG (2015). Multivariable Mendelian randomization: the use of pleiotropic genetic variants to estimate causal effects. Am J Epidemiol.

[24] Burgess S, Bowden J, Fall T, Ingelsson E, Thompson SG (2017). Sensitivity Analyses for Robust Causal Inference from Mendelian Randomization Analyses with Multiple Genetic Variants. Epidemiology.

[25] Slatkin M (2008). Linkage disequilibrium--understanding the evolutionary past and mapping the medical future. Nat Rev Genet.

[26] Hamer D, Sirota L (2000). Beware the chopsticks gene. Mol Psychiatry.

[27] Price AL, Patterson NJ, Plenge RM, Weinblatt ME, Shadick NA, Reich D (2006). Principal components analysis corrects for stratification in genome-wide association studies. Nat Genet.

[28] Price AL, Zaitlen NA, Reich D, Patterson N (2010). New approaches to population stratification in genome-wide association studies. Nat Rev Genet.

[29] Bouaziz M, Ambroise C, Guedj M (2011). Accounting for population stratification in practice: a comparison of the main strategies dedicated to genome-wide association studies. PLoS One.

[30] Schmidt AF, Finan C, Gordillo-Marañón M (2020). Genetic drug target validation using Mendelian randomisation. Nature Communications.

[31] Wensley F, Gao P, Burgess S (2011). Association between C reactive protein and coronary heart disease: mendelian randomisation analysis based on individual participant data. Bmj.

[32] Davey Smith G, Hemani G (2014). Mendelian randomization: genetic anchors for causal inference in epidemiological studies. Human Molecular Genetics.

[33] Rühlemann MC, Hermes BM, Bang C (2021). Genome-wide association study in 8,956 German individuals identifies influence of ABO histo-blood groups on gut microbiome. Nature Genetics.

[34] Burgess S, Scott RA, Timpson NJ, Davey Smith G, Thompson SG, EPIC-InterAct Consortium (2015). Using published data in Mendelian randomization: a blueprint for efficient identification of causal risk factors. European Journal of Epidemiology.

[35] Titova OE, Michaëlsson K, Larsson SC (2020). Sleep Duration and Stroke. Stroke.

[36] Liao W-Z, Zhou Z-Y, Lin Z-K (2023). Coffee consumption and periodontitis: a Mendelian Randomization study. Genes & Nutrition.

[37] Rhee Y, Choi Y, Park J, Park HR, Kim K, Kim YH (2022). Association between coffee consumption and periodontal diseases: a systematic review and meta-analysis. BMC Oral Health.

[38] Au Yeung SL, Gill D (2024). Concerns over using the Mendelian randomization design to investigate the effect of air pollution. Science of The Total Environment.

[39] Kinane DF, Stathopoulou PG, Papapanou PN (2017). Periodontal diseases. Nature Reviews Disease Primers.

[40] Marruganti C, Romandini M, Gaeta C (2023). Healthy lifestyles are associated with a better response to periodontal therapy: A prospective cohort study. J Clin Periodontol.

[41] Marruganti C, Shin HS, Sim SJ, Grandini S, Laforí A, Romandini M (2023). Air Pollution as a Risk Indicator for Periodontitis. Biomedicines.

[42] Darby I (2022). Risk factors for periodontitis & peri-implantitis. Periodontol 2000.

[43] Cekici A, Kantarci A, Hasturk H, Van Dyke TE (2014). Inflammatory and immune pathways in the pathogenesis of periodontal disease. Periodontology 2000.

[44] Corana M, Baima G, Iaderosa G (2024). Salivary Proteomics for Detecting Novel Biomarkers of Periodontitis: A Systematic Review. J Periodontal Res.

[45] Muth KN, Rech J, Losch FO, Hoerning A (2023). Reversing the Inflammatory Process—25 Years of Tumor Necrosis Factor-α Inhibitors. Journal of Clinical Medicine.

[46] Breseghello I, Rosalen PL, Bruzadelli RFD Phthalocyanine derivative attenuates TNF-α production in macrophage culture and prevents alveolar bone loss in experimental periodontitis. Journal of Periodontal Research.

[47] Michalowicz BS, Aeppli D, Virag JG (1991). Periodontal Findings in Adult Twins. Journal of Periodontology.

[48] Gao C, Iles M, Larvin H (2024). Genome-wide association studies on periodontitis: A systematic review. PLOS ONE.

[49] Burgess S, Labrecque JA (2018). Mendelian randomization with a binary exposure variable: interpretation and presentation of causal estimates. European Journal of Epidemiology.

[50] Angrist JD, Krueger AB (1991). Does Compulsory School Attendance Affect Schooling and Earnings?*. The Quarterly Journal of Economics.

[51] Angrist JD, Lavy V (1999). Using Maimonides' Rule to Estimate the Effect of Class Size on Scholastic Achievement*. The Quarterly Journal of Economics.

[52] Slob EAW, Burgess S (2020). A comparison of robust Mendelian randomization methods using summary data. Genet Epidemiol.

[53] Bowden J, Davey Smith G, Burgess S (2015). Mendelian randomization with invalid instruments: effect estimation and bias detection through Egger regression. International Journal of Epidemiology.

[54] Hartwig FP, Davey Smith G, Bowden J (2017). Robust inference in summary data Mendelian randomization via the zero modal pleiotropy assumption. International Journal of Epidemiology.

[55] Verbanck M, Chen CY, Neale B, Do R (2018). Detection of widespread horizontal pleiotropy in causal relationships inferred from Mendelian randomization between complex traits and diseases. Nat Genet.

[56] Papapanou PN, Sanz M, Buduneli N (2018). Periodontitis: Consensus report of workgroup 2 of the 2017 World Workshop on the Classification of Periodontal and Peri-Implant Diseases and Conditions. Journal of Clinical Periodontology.

[57] Tran DT, Gay I (2013). Assessing periodontitis in populations: a systematic review of the validity of partial-mouth examination protocols. J Clin Periodontol.

[58] Lindhe J, Westfelt E, Nyman S, Socransky SS, Haffajee AD (1984). Long-term effect of surgical/non-surgical treatment of periodontal disease. J Clin Periodontol.

[59] Goodson JM, Haffajee AD, Socransky SS (1984). The relationship between attachment level loss and alveolar bone loss. J Clin Periodontol.

[60] Bassett E, Broadbent J, Gill D, Burgess S, Mason AM (2024). Inconsistency in UK Biobank Event Definitions From Different Data Sources and Its Impact on Bias and Generalizability: A Case Study of Venous Thromboembolism. American Journal of Epidemiology.

[61] Baima G, Shin HS, Arrica M, Laforí A, Cordaro M, Romandini M (2023). The co-occurrence of the two main oral diseases: periodontitis and dental caries. Clin Oral Investig.

[62] Romandini P, Marruganti C, Romandini WG, Sanz M, Grandini S, Romandini M (2024). Are periodontitis and dental caries associated? A systematic review with meta-analyses. J Clin Periodontol.

[63] Shungin D, Haworth S, Divaris K (2019). Genome-wide analysis of dental caries and periodontitis combining clinical and self-reported data. Nature Communications.

[64] Verma A, Huffman JE, Rodriguez A (2024). Diversity and scale: Genetic architecture of 2068 traits in the VA Million Veteran Program. Science.

[65] Bevilacqua L, Navarra CO, Pirastu N, Lenarda RD, Gasparini P, Robino A (2018). A genome-wide association study identifies an association between variants in EFCAB4B gene and periodontal disease in an Italian isolated population. Journal of Periodontal Research.

[66] Hong KW, Shin MS, Ahn YB, Lee HJ, Kim HD (2015). Genomewide association study on chronic periodontitis in Korean population: results from the Yangpyeong health cohort. Journal of clinical periodontology.

[67] Munz M, Richter GM, Loos BG (2019). Meta-analysis of genome-wide association studies of aggressive and chronic periodontitis identifies two novel risk loci. European Journal of Human Genetics.

[68] Schaefer AS, Richter GM, Nothnagel M (2010). A genome-wide association study identifies GLT6D1 as a susceptibility locus for periodontitis. Human molecular genetics.

[69] Shaffer JR, Polk DE, Wang X (2014). Genome-Wide Association Study of Periodontal Health Measured by Probing Depth in Adults Ages 18− 49 years. G3: Genes, Genomes, Genetics.

[70] Shimizu S, Momozawa Y, Takahashi A (2015). A genome-wide association study of periodontitis in a Japanese population. Journal of dental research.

[71] Tegelberg P, Leppilahti JM, Ylöstalo A (2021). Genome-wide association study of periodontal pocketing in Finnish adults. BMC oral health.

[72] Munz M, Willenborg C, Richter GM (2017). A genome-wide association study identifies nucleotide variants at SIGLEC5 and DEFA1A3 as risk loci for periodontitis. Human molecular genetics.

[73] de Coo A, Cruz R, Quintela I (2021). Genome-wide association study of stage III/IV grade C periodontitis (former aggressive periodontitis) in a Spanish population. Journal of Clinical Periodontology.

[74] Petty LE, Silva R, de Souza LC (2023). Genome-wide association study identifies novel risk loci for apical periodontitis. Journal of endodontics.

[75] Divaris K, Monda KL, North KE (2013). Exploring the genetic basis of chronic periodontitis: a genome-wide association study. Human molecular genetics.

[76] Feng P, Wang X, Casado PL (2014). Genome wide association scan for chronic periodontitis implicates novel locus. BMC oral health.

[77] Sanders A, Sofer T, Wong Q (2017). Chronic periodontitis genome-wide association study in the Hispanic Community Health Study/Study of Latinos. Journal of dental research.

[78] Teumer A, Holtfreter B, Völker U (2013). Genome-wide association study of chronic periodontitis in a general German population. Journal of clinical periodontology.

[79] Kurki MI, Karjalainen J, Palta P (2023). FinnGen provides genetic insights from a well-phenotyped isolated population. Nature.

[80] Nogawa S, Morishita S, Saito K, Kato H (2024). Genome-wide association meta-analysis identifies two novel loci associated with dental caries. BMC Oral Health.

[81] De Almeida SD, Richter GM, de Coo A (2024). A genome-wide association study meta-analysis in a European sample of stage III/IV grade C periodontitis patients ≤35 years of age identifies new risk loci. Journal of Clinical Periodontology.

[82] Papapanou PN, Sanz M, Buduneli N (2018). Periodontitis: Consensus report of workgroup 2 of the 2017 World Workshop on the Classification of Periodontal and Peri-Implant Diseases and Conditions. J Periodontol.

[83] Armitage GC (1999). Development of a classification system for periodontal diseases and conditions. Ann Periodontol.

[84] Page RC, Eke PI (2007). Case definitions for use in population-based surveillance of periodontitis. J Periodontol.

[85] Eke PI, Page RC, Wei L, Thornton-Evans G, Genco RJ (2012). Update of the case definitions for population-based surveillance of periodontitis. J Periodontol.

[86] Liu L, Wang T, Duan C (2024). Genetically Supported Drug Targets and Dental Traits: A Mendelian Randomization Study. Journal of Dental Research.

[87] Munafò MR, Higgins JPT, Davey Smith G (2021). Triangulating Evidence through the Inclusion of Genetically Informed Designs. Cold Spring Harbor Perspectives in Medicine.

[88] Casanova L, Hughes FJ, Preshaw PM (2014). Diabetes and periodontal disease: a two-way relationship. British Dental Journal.

[89] Wang Y, Chu T, Gong Y (2022). Mendelian randomization supports the causal role of fasting glucose on periodontitis. Frontiers in Endocrinology.

[90] Leite FRM, Nascimento GG, Scheutz F, López R (2018). Effect of Smoking on Periodontitis: A Systematic Review and Meta-regression. American Journal of Preventive Medicine.

[91] Wang J, Lv J, Wang W, Jiang X (2016). Alcohol consumption and risk of periodontitis: a meta-analysis. Journal of Clinical Periodontology.

[92] Baumeister S-E, Freuer D, Nolde M (2021). Testing the association between tobacco smoking, alcohol consumption, and risk of periodontitis: A Mendelian randomization study. Journal of Clinical Periodontology.

[93] Baumeister SE, Listl S, Holtfreter B, Nascimento GG, Leite FRM (2024). Causal Effect of Smoking and Cessation on Tooth Loss. J Clin Periodontol.

[94] Boillot A, El Halabi B, Batty GD, Rangé H, Czernichow S, Bouchard P (2011). Education as a Predictor of Chronic Periodontitis: A Systematic Review with Meta-Analysis Population-Based Studies. PLOS ONE.

[95] Baumeister SE, Freuer D, Baurecht H (2022). Understanding the consequences of educational inequalities on periodontitis: a Mendelian randomization study. Journal of Clinical Periodontology.

[96] Burgess S, Woolf B, Mason AM, Ala-Korpela M, Gill D (2024). Addressing the credibility crisis in Mendelian randomization. BMC Med.

