# Supplementary material for: A Principled Framework for Mendelian Randomization in Oral Health Research
Source: J Periodontal Res. Author manuscript; Available in PMC 2025 Aug 14. (PMC7618009; doi:10.1111/jre.13411)
Supplement: Box 1 [file EMS207592-supplement-Box_1.docx]

**Box 1.** A summary of the steps involved in a MR study (adapted from Burgess et al.).^2^

| **1. Define the motivation and scope**   - State clearly the exposure(s) and outcome(s), and the causal hypotheses to be tested   **2. Choose data sources**   - Identify appropriate datasets for selecting genetic variants, and estimating associations with the exposure(s) and outcome(s)   **3. Select genetic variants**   - Identify genetic variants associated with the exposure that plausibly satisfy the core instrumental variable assumptions (relevance, exchangeability, and exclusion restriction)   **4. Harmonize genetic data (for two-sample investigations)**   - Ensure genetic variants are consistently aligned across datasets for exposure and outcome, accounting for allele frequency and strand orientation   **5. Perform primary analysis**   - The primary analysis represents the initial assessment of evidence for a causal effect of the exposure on the outcome   **6. Conduct sensitivity analyses**   - Sensitivity analyses are performed to “stress test” the primary analysis result: to see how robust it is to various conditions, including violations of the instrumental variable assumptions (for example, alternative statistical methods, analyses excluding certain genetic variants, colocalization, assessment of positive/negative controls)   **7. Explore supplementary analyses**   - Supplementary analyses are performed to better understand the nature of the causal relationship (for example, is it similar in different populations or population subgroups?)   **8. Present findings transparently**   - Data and results should be presented with enough information to allow readers to assess the plausibility of the analysis and its assumptions   **9. Interpret results with appropriate caution**   - Results should be presented and interpreted with appropriate caution, and compared with evidence from the wider literature in a triangulation framework |
| --- |
